# The Multipartite Mitochondrial Genome of *Liposcelis bostrychophila*: Insights into the Evolution of Mitochondrial Genomes in Bilateral Animals

**DOI:** 10.1371/journal.pone.0033973

**Published:** 2012-03-30

**Authors:** Dan-Dan Wei, Renfu Shao, Ming-Long Yuan, Wei Dou, Stephen C. Barker, Jin-Jun Wang

**Affiliations:** 1 Key Laboratory of Entomology and Pest Control Engineering, College of Plant Protection, Southwest University, Chongqing, China; 2 School of Chemistry and Molecular Biosciences, The University of Queensland, Brisbane, Queensland, Australia; 3 School of Science, Education and Engineering, University of the Sunshine Coast, Maroochydore, Queensland, Australia; University of Iceland, Iceland

## Abstract

Booklice (order Psocoptera) in the genus *Liposcelis* are major pests to stored grains worldwide and are closely related to parasitic lice (order Phthiraptera). We sequenced the mitochondrial (mt) genome of *Liposcelis bostrychophila* and found that the typical single mt chromosome of bilateral animals has fragmented into and been replaced by two medium-sized chromosomes in this booklouse; each of these chromosomes has about half of the genes of the typical mt chromosome of bilateral animals. These mt chromosomes are 8,530 bp (mt chromosome I) and 7,933 bp (mt chromosome II) in size. Intriguingly, mt chromosome I is twice as abundant as chromosome II. It appears that the selection pressure for compact mt genomes in bilateral animals favors small mt chromosomes when small mt chromosomes co-exist with the typical large mt chromosomes. Thus, small mt chromosomes may have selective advantages over large mt chromosomes in bilateral animals. Phylogenetic analyses of mt genome sequences of Psocodea (i.e. Psocoptera plus Phthiraptera) indicate that: 1) the order Psocoptera (booklice and barklice) is paraphyletic; and 2) the order Phthiraptera (the parasitic lice) is monophyletic. Within parasitic lice, however, the suborder Ischnocera is paraphyletic; this differs from the traditional view that each suborder of parasitic lice is monophyletic.

## Introduction

The mitochondrial (mt) genomes of bilateral animals typically consist of a single circular chromosome that is 13 to 20 kb in size and contains 37 genes: 13 protein-coding genes, 22 transfer RNA genes and two ribosomal RNA genes [1]. Exceptions to the typical mt chromosomes, however, have been discovered in the past decade. Mitochondrial genomes that consist of multiple chromosomes, i.e. multipartite mt genomes, have been found in mesozoa, nematodes, rotifers and parasitic lice [2]–[7]. The number of chromosomes in a multipartite mt genome varies from two in the rotifer, *Brachionus plicatilis* [5], to 20 in the human body louse, *Pediculus humanus* [6]. Each chromosome in a multipartite mt genome is circular and comprises a coding region and a non-coding region. In an extreme case in the human body louse, an mt chromosome contains only a single tRNA gene [6]. Sequences of the non-coding regions, or segments in the non-coding regions, are often highly conserved among all of the chromosomes in a multipartite mt genome, indicating the functional roles of non-coding regions, potentially in mt genome replication and gene transcription [2], [5], [6]. Different chromosomes in a multipartite mt genome are in unequal abundance [5]; furthermore, the relative abundance of an mt chromosome varies at different developmental stages [8].

Multipartite mt genomes (circular or linear) are not unique to bilateral animals, and have been found in plants [9], fungi [10], cnidarians [11], and various lineages of protists, including algae [12], ciliates [13], flagellates [14], and ichthyosporeans [15]. The multipartite mt genomes of non-bilateral animals exhibit more diversity than their counterparts in bilateral animals. For instance, the mt genome of the box jellyfish, *Alatina moseri*, consists of eight linear chromosomes [16]. The mitochondrial genome of *Diplonema papillatum*, a free-living diplonemids, is composed of more 100 circular chromosomes; some of these chromosomes contain only a segment of genes [14], [17].

Why are mt genomes in pieces? Several possibilities have been raised, including Darwinian selective advantage, random genetic drift [18], loss of mitochondrial single-strand binding protein (mtSSB) [7], blood-feeding [6] and parasitic life-style [19]. None of these possibilities, however, has been investigated experimentally. Furthermore, two mechanisms have been proposed to explain how multipartite mitochondrial genomes are generated: 1) intramolecular homologous recombination [20]; and gene deletions [21]. None of these mechanisms, however, could explain why multipartite mt genomes should replace the typical single-chromosome mt genomes.

Booklice (order Psocoptera) in the genus *Liposcelis* are major pests to stored grains worldwide and are closely related to parasitic lice of birds and mammals (order Phthiraptera). The monophyly of the order Phthiraptera has been questioned recently [22], [23]. There are two contradictory hypotheses: 1) the parasitic lice (order Phthiraptera) are monophyletic and booklice of the genus *Liposcelis* (order Psocoptera), are the sister-group to the parasitic lice [24], [25]; and 2) the parasitic lice are paraphyletic and the booklice of the genus *Liposcelis* are the sister-group to one of the four suborders of the parasitic lice, the Amblycera [22], [23], [26]. Monophyly of another suborder of the parasitic lice, the Ischnocera, has also been questioned: some species in the Ischnocera have been shown to be more closely related to blood-sucking lice in the suborder Anoplura than to other Ischnocera species [7], [27].

Mitochondrial genome sequences have been used extensively in inferring phylogenetic relationships among insects at different taxonomic levels [28]–[30]. Thus far, complete mt genome sequences have been determined for more than 300 species of insects (available at http://www.ncbi.nlm.nih.gov). Only one of these species, a barklouse, Lepidopsocid sp., however, is from the order Psocoptera [31]; no booklice have been sequenced for complete mitochondrial genomes. In this study, we sequenced the mt genome of the booklouse, *Liposcelis bostrychophila* Badonnel (Psocoptera: Liposcelididae). Here, we report the features of the mt genome of *L. bostrychophila*, and the phylogenetic relationships among the major lineages of the Psocodea, i.e. booklice, barklice and parasitic lice, inferred from mt genome sequences.

## Materials and Methods

### Ethics Statement

No specific permits were required for the described field studies, and no specific permissions were required for these locations/activities. We confirm that these locations are not privately-owned or protected in any way and the field studies did not involve endangered or protected species.

### Sample collection, DNA extraction, PCR amplification and sequencing

**Figure 1:**
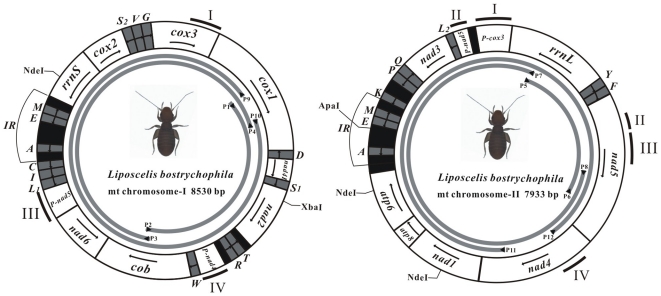
The mitochondrial genome of *Liposcelis bostrychophila*. The directional transcription is indicated with arrows. Protein-coding and ribosomal genes are shown in white with standard abbreviations. tRNA genes are abbreviated by a single letter with dark gray except the 2 serine and 2 leucine tRNAs: S1 = AGN, S2 = UCN, L1 = CUN, and L2 = UUR. The non-coding regions >50 bp are indicated in black. IR, an identical region in mt chromosome I and mt chromosome II. The four pseudogenes and their corresponding position in the putatively functional genes are indicated by the number I, II, III, and IV. The cut sites of restriction enzymes used for verification of the multipartite mitochondrial genome, NdeI, XbaI, NdeI, and ApaI are shown. Arrows and gray curves indicate primers and PCR fragment, respectively. See Table 1 for PCR primers. The figures are approximately to scale.

*L. bostrychophila* were collected at grain storage facilities from six localities in five provinces in China (Table S1). These insects were identified to species by morphology [32] and internal transcribed spacer (ITS) sequences [33]. Total DNA was extracted from *L. bostrychophila* by the SDS method [34]. For real-time PCR, the total DNA was also extracted using DNeasy Blood & Tissue Kit (QIAGEN) from the Beibei strain of *L. bostrychophila*. Fragments of *cox1*and *cob* were amplified by PCR with conserved insect primers [35]. PCR products were purified with Gel Extraction Mini kits (Watson Biotechnologies). Purified PCR products were ligated into pGEM-T Easy vectors (Promega, Madison, WI) and introduced into *Escherichia coli* (T1, Transgen) followed by ampicillin selection. Positive clones were sequenced with a 3730, ABI Applied Biosystems sequencer. Then, species-specific primers were designed for long-PCR from *cox1* to *cob* sequences. Long PCR reactions were carried out in a total volume of 25 µl, utilizing 1 µl of DNA, 1 µl each of two primers (10 µM; P1–P2, P3–P4; Figure 1), 4 µl of dNTPs (each 2.5 mM), 2.5 µl MgCl_2_ (25 mM), 2.5 µl of 10× LA PCR reaction buffer, 12.75 µl ddH_2_O and 0.25 µl LA Taq DNA polymerase (5 U/µl, Takara). This was performed on Bio-RAD S1000™ thermal cyclers and the PCR conditions were: 1 min at 96°C, 37 cycles of 40 sec at 96°C, 50 sec at 60°C and 9 min at 68°C, followed by 15 min at 68°C. The two overlapping PCR fragments (P1–P2, and P3–P4; Figure 1) were directly sequenced using primer walking method by the Beijing Genomics Institute (BGI) at Shenzhen, China. The nucleotide sequences were proof-read and assembled into a contig with SeqMan (DNAStar). Initially, only seven protein-coding genes were found in the 8,530 bp contig that was amplified by long-PCR primers from *cox1* and *cob.* So, we amplified and sequenced fragments of two other mitochondrial genes, *rrnL* and *nad5*, which were not in the first contig, with conserved insect mitochondrial primers [35]. Two overlapping fragments were amplified by long-PCR with the primers designed from *rrnL* and *nad5* sequences (P5–P6, P7–P8; Figure 1). These two fragments were sequenced and a contig was assembled; six other protein-coding genes were found in this contig. Sequences of the primers used in this study are presented in Table 1.

**Table 1:** Primers used for PCR amplification in this study.

Gene	Primer	Primer sequence (5′- 3′)	Product size (bp)
*cox1*	UEA3	TATAGCATTCCCACGAATAAATAA	UEA3-UEA8: 1016
*cox1*	UEA8	AAAAATGTTGAGGGAAAAATGTTA	
*cob*	CBF1	TATGTACTACCATGAGGACAAATATC	CBF1-CBR1: 485
*cob*	CBR1	ATTACACCTCCTAATTTATTAGGAAT	
*rrnL*	16Sar	CGCCTGTTTAACAAAAACAT	16Sar-16Sbr: 490
*rrnL*	16Sbr	CCGGTCTGAACTCAGATCACGT	
*nad5*	N5-J7077	TTAAATCCTTWGARTAAAAYCC	J7077-N7793: 733
*nad5*	N5-N7793	TTAGGTTGRGATGGNYTAGG	
*cox1*	P1	GAGGGGGGGACCCAATTCTCTTTCAATATT	P1–P2: 3173
*cob*	P2	GAAATGATAGGAATCGTGTTAGTGTGGGAT	
*cob*	P3	ATCCCACACTAACACGATTCCT	P3–P4: 5853
*cox1*	P4	ACATAGTGGAAGTGGGCAACAACAT	
*cox1*	P9	CTATGTTGTTGCCCACTTCCACTATGTTC	P9–P10: 8098
*cox1*	P10	CCCCCTCCAGATGGATCAAAGAATGAAGTT	
*rrnL*	P5	TTAATTCAACATCGAGGTCGCAATCACAAG	P5–P6: 2099
*nad5*	P6	TGGAAGCCCCATTATTGAGCAT	
*nad5*	P7	CTGGTGTGTATCTCCTCTATCG	P7–P8: 6244
*rrnL*	P8	CTTGTGATTGCGACCTCGATGTTGAATTAA	
*nad4*	P11	TTATGTTCGTGTCAAAACCCG	P11–P12: 7394
*nad4*	P12	TAGTCGTTCATACTGTTTCCCTC	
*cob*	qcobF	TTGGGGTCGCTTCTCGGGCT	qcobF-qcobR: 201
*cob*	qcobR	TCCGACATGGGCGTAGCAGA	
*nad1*	qnad1F	TAGTATTATCCTTCTTTATTGGC	qnad1F- qnad1R: 157
*nad1*	qnad1R	ACAATGGTTAGGGGGAGGTAGAT	
*Nuclear gene*	*β-Actin*S	CACGGTATCGTCACCAACTG	*β-Actin*S-*β-Actin*A: 207
	*β-Actin*A	AGACAATACGGCTTGGATGG	

### Sequence analysis

All of the typical protein-coding genes, except *atp8* and *nad4L*, were identified by BLAST searches of NCBI database. Two genes *atp8* and *nad4L* were identified by comparison of putative amino acid sequences and hydrophilicity profiles with those of other insects in NCBI database. A highly conserved amino acid sequence motif, MPQMAPL, at the N-terminal of *ATP8* protein of insects was also used to help find *atp8* gene. Hydrophilicity profiles were generated with MacVector [36] (Figure S1). rRNA genes were identified by BLAST searches of NCBI database. All of the typical tRNA genes of animals, except *trnE* and *trnR*, were identified by their clover-leaf secondary structure using tRNAscan-SE [37] and ARWEN [38]. *trnE* and *trnR* results were identified by manual inspection of potential anticodon sequences and by manual sequence alignment with *trnE* and *trnR* of other barklice and parasitic lice. Secondary structures in the largest non-coding regions (putative control regions) were predicted with program RNAstructure v. 5.0 [39]. Base compositions and codon usage were calculated with Mega 5 [40].

### PCR verification of the multipartite mitochondrial genome of *L. bostrychophila* and quantification of the two mitochondrial chromosomes

**Figure 2:**
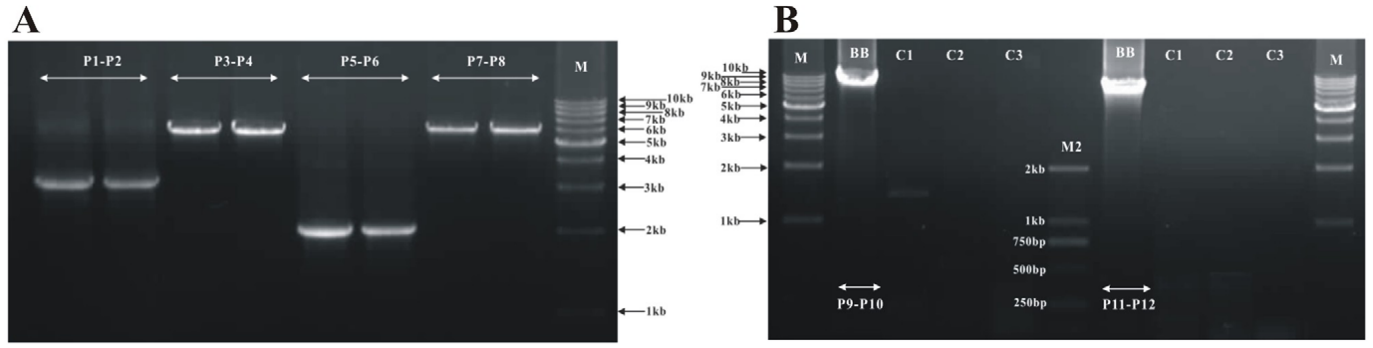
PCR amplification of mitochondrial DNA from Beibei strain of *Liposcelis bostrychophila.* (A) Initial Long-PCR amplification of four fragments for the mitochondrial genome of *L. bostrychophila*. (B) PCR tests to verify the multipartite mt genomes in the Beibei strains of *L. bostrychophila*. Lane C1, negative control without the forward primer P9 or P11; lane C2, negative control without the reverse primer P10 or P12; lane C3, negative control without the DNA template. Lane M: 1 kb marker (TaKaRa). Lane M2: DL2000 bp marker (TaKaRa). “P1–P2”, the product of PCR with primers P1 and P2, etc. Primer names in Table 1.

We verified the size and the circular nature of the two contigs by PCR with two pairs of outbound primers: P9 and P10 in the *cox1* gene, and P11 and P12 in the *nad4* gene (Figure 1 and Figure 2). The two contigs are hereafter referred to as mt chromosome I and chromosome II. We quantified the relative copy numbers of chromosome I and chromosome II of the Beibei strain of *L. bostrychophila* by real-time PCR with an Mx3000P thermal cycler (Stratagene). Each PCR reaction contained 1 µl of genomic DNA, 12.5 µl SYBR® Premix Ex Taq™ II (Takara) and 0.2 mM of each primer. A pair of primers, qcobF and qcobR, were designed from *cob* gene for mt chromosome I; another pair, qnad1F and qnad1R, were designed from *nad1* gene for mt chromosome II (Table 1). The real-time PCR conditions were: 95°C for 1 min, followed by 40 cycles of 95°C for 10 sec, 60°C for 30 sec and 72°C for 30 sec. A melting curve analysis from 55°C to 95°C was applied to all reactions to ensure consistency and specificity of the amplified product. A nuclear *β*-*Actin* gene was used as a reference [41]. The amplification efficiency of real-time PCR was calculated by the MxPro 4.01 software for Mx3000P (Stratagene) based on dilution curves. The amount of target gene relative to the reference gene was calculated by the 2^−ΔCt^ method [42]. Real-time PCR was repeated three times with total DNA extracted from the two different methods.

### Phylogenetic analysis of the mitochondrial genome sequences of booklice, barklice and parasitic lice (i.e. Psocodea)

We inferred phylogenies using the mt genome sequences of the booklouse: *L. bostrychophila* (Psocoptera: Troctomorpha) (this paper), the barklouse, Lepidopsocid sp. RS-2001 (Psocoptera: Trogiomorpha) [31], and the parasitic lice: *Bothriometopus macrocnemis* (Phthiraptera: Ischnocera) [43], *Campanulotes bidentatus* (Phthiraptera: Ischnocera) [44], *Heterodoxus macropus* (Phthiraptera: Amblycera) [45] and *P. humanus* (Phthiraptera: Anoplura) [6]. *Pachypsylla venusta* (Hemiptera: Psylloidea) [46] was used as an outgroup. It was difficult to align the putative amino acid and the nucleotide sequences of *atp6*, *atp8*, *nad4L*, and the nucleotide sequences of the tRNA genes because the length of these genes varied substantially among the five species listed above. So, we excluded *atp6*, *atp8*, *nad4L* and the tRNA genes from our phylogenetic analyses.

Putative amino acid and nucleotide sequences were aligned using Clustal X with the default parameters [47]. Alignments were then imported into the Gblocks server (Http://molevol.cmima.csic.es/castresana/Gblocks_server.html) [48] to remove poorly aligned sites. Nucleotide sequences of the protein-coding genes were tested for substitution saturation using DAMBE 5.0.59 [49]. All of the protein-coding genes, except *nad2* and *nad3*, passed this test: the second and third codon positions of *nad2* were excluded, whereas the first and the third positions of *nad3* were excluded from our phylogenetic analyses. Phylogenies were inferred from two alignments: (1) the putative amino acid sequences of 10 protein-coding genes (*cox1*, *cox2*, *cox3*, *nad1*, *nad2*, *nad3*, *nad4*, *nad5*, *nad6*, and *cob*), and (2) the nucleotide sequences of the same 10 protein-coding genes plus the two rRNA genes (*rrnS*, *rrnL*). The amino acid and nucleotide sequence alignments had 2,133 and 10,173 positions, respectively (includes alignment gaps). The best-fit model for the amino acid sequence alignment was determined with MEGA 5 [40]: the RtREV model was selected. For the nucleotide sequence alignment, jModelTest 0.1.1 [50] was used to find a suitable model for nucleotide substitution using the Akaike Information Criterion: the GTR+I+G model were chosen. Phylogenies were inferred by Bayesian Inference (BI) with MrBayes v3.12 [51], Maximum Likelihood (ML) with PhyML 3.0 (http://www.atgcmontpellier.fr/phyml/) [52], and Neighbor-Joining (NJ) with MEGA 5 [40]. For Bayesian Inference, four independent Markov chains were simultaneously run for 500,000 generations with a heating scheme (temp = 0.5). Trees were sampled every 100 generations (samplefreq = 100) and the first 20% of the generations were discarded as burn-in and the remaining samples were used to compute the consensus tree. Stationarity was considered to be reached when the average standard deviation of split frequencies was below 0.01 [53]. There were 100 bootstrap replicates in ML analysis and 1000 replicates for NJ analysis.

## Results

### The Beibei strain of *L. bostrychophila* has a multipartite mitochondrial genome

Two overlapping fragments, 3.1 and 5.8 kb in size, were amplified by PCR with the primer pairs P1and P2 on the one hand, P3 and P4 on the other hand (Figure 1 and Figure 2). We sequenced these two PCR fragments; the sequences were assembled into a circle, 8,530 bp in size; we called this circle mt chromosome I hereafter. Unexpectedly, mt chromosome I has only half of the genes that are typically found in the mt genome of a bilateral animal (Figure 1).Thus, we sought the other mt genes with primers that were anchored in *rrnL* and *nad5* (P5, P6 and P7, P8, Figure 1). PCR with these primers amplified a 2 kb and a 6.2 kb fragment, respectively (Figure 2). The sequences of these two fragments were assembled into another circle, 7,933 bp in size; we called this circle mt chromosome II (Figure 1). Of the 35 mt genes typical of the mt genome of bilateral animals, 22 genes were found only in mt chromosome I whereas 16 genes were only in mt chromosome II (Figure 1, Table S2 and S3). Intriguingly, three tRNA genes, *trnA*, *trnE* and *trnM*, were found in both chromosomes. The longest non-coding region (485 bp), was present in both chromosomes; we called this non-coding region the control region hereafter (Figure 2). Sequences of *trnA*, *trnE*, *trnM* and the control region are identical between mt chromosome I and mt chromosome II. We could not find two tRNA genes, *trnH* and *trnN*, in either chromosome I or chromosome II. Sequences of mt chromosome I and mt chromosome II were in GenBank under accession numbers JN645275 and JN645276.

### General features of the multipartite mitochondrial genome of the Beibei strain of *L. bostrychophila*

**Figure 3:**
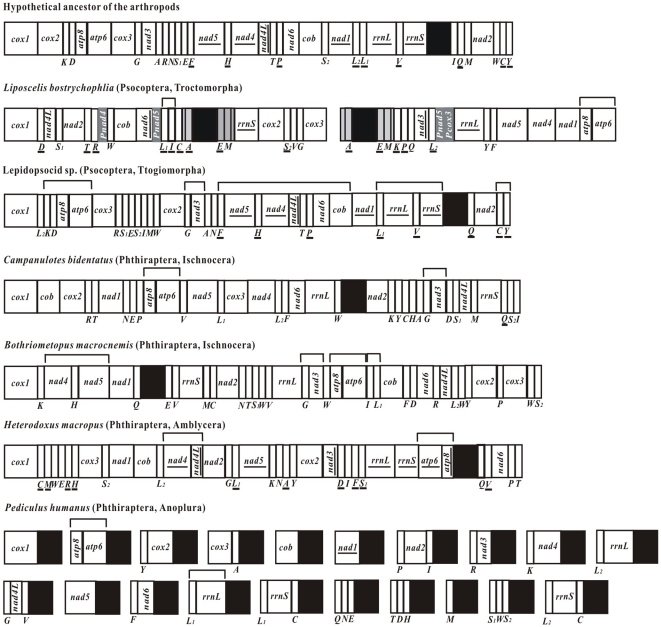
Arrangement of mitochondrial genes in the booklouse, barklouse and parasitic lice that have been sequenced and the gene-arrangement of the hypothetical ancestor of the arthropods. Circular genomes have been arbitrarily linearized for ease of comparison. Gene names are the standard abbreviations used in the present study. tRNA genes are designated by the single letter according to the IPUC-IUB one-letter amino acid codes. Genes which are underlined are encoded on the opposite strand to the majority of genes in that particular genome. Black boxes represent putative control regions. Shared gene-boundaries are labeled with square “brackets” above each genome.

Genes were on both strands of the mt chromosomes of the Beibei strain of *L. bostrychophila*. For mt chromosome I, six protein-coding genes and five tRNA genes were on one strand whereas one protein-coding gene, one rRNA gene and nine tRNA genes were on the other strand (Figure 1). For mt chromosome II, five protein-coding genes and four tRNA genes were on one strand whereas one protein-coding gene, one rRNA gene and five tRNA genes were on the other strand (Figure 1). The gene-boundary *trnI*-*trnL1* is present in *L. bostrychophila* and the parasitic screamer louse, *B. macrocnemis* (Ischnocera); this boundary is derived for insects and appears to have evolved independently in the lineages that led to *L. bostrychophila* and *B. macrocnemis* (Figure 3). The only gene-boundary that *L. bostrychophila* shares with all other insects is *atp8*-*atp6*, which is ancestral to insects (Figure 3).

**Figure 4:**
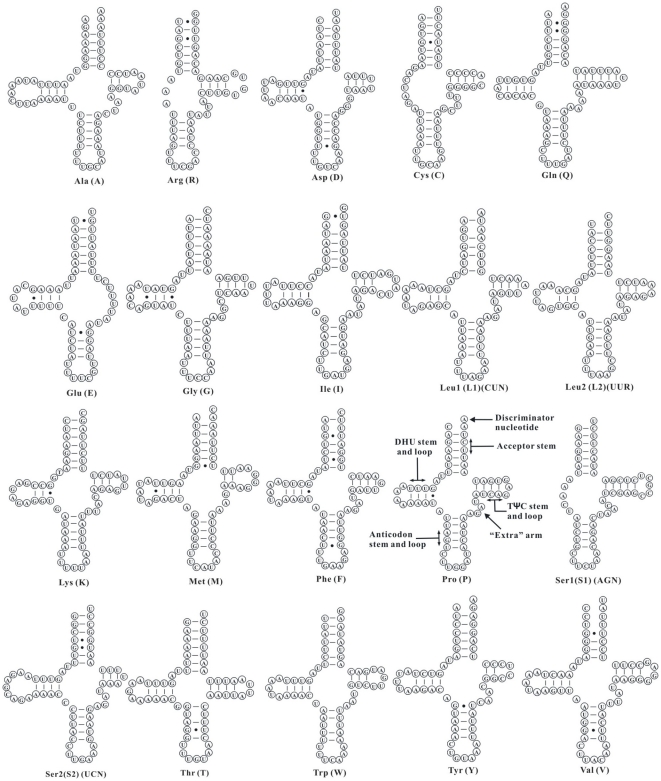
Putative secondary structures of the 20 tRNA genes identified in the mitochondrial genome of *Liposcelis bostrychophila*. Bars indicate Watson-Crick base pairings, and dots between G and U pairs mark canonical base pairings appearing in RNA.

**Figure 5:**
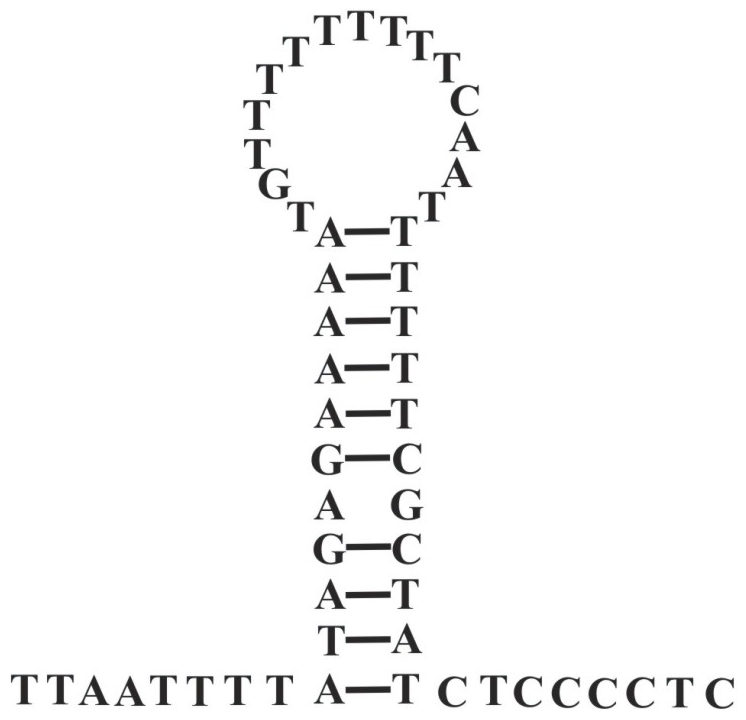
A putative stem-loop in the 485 bp (NCRI-4 = NCRII-3) identity non-coding regions of two mitochondrial chromosomes in *Liposcelis bostrychophila*.

Sixteen of the 20 tRNA genes in the Beibei strain of *L. bostrychophila* have the conventional clover-leaf secondary structures (Figure 4). Three tRNA genes, *trnR*, *trnC* and *trnS_1_*, lack the sequences for D-arms whereas *trnE* lacks the sequence for a T-arm. There is a stem-and-loop with a poly-T stretch in the loop in the control regions of both mt chromosomes (Figure 5); this may contain the sites of initiation of replication and transcription. There are two putative pseudogenes in mt chromosome I: P-nad4 (IV, 216 bp) and P-nad5 (III, 207 bp); and two putative pseudogenes in mt chromosome II: P-cox3 (I, 322 bp) and P-nad5 (II, 108 bp) (Figure 1 and Figure S2). These putative pseudogenes have identical or near-identical sequences to parts of their full-length counterparts. The A+T content of mt chromosome I and mt chromosome II were 67.78% and 69.54%, respectively. The nucleotide composition, AT-skew, and GC-skew between the two chromosomes and among the genes were summarized in Table 2, Table S2, and Table S3. The codon usages for the 13 mt protein-coding genes of *L. bostrychophila* were summarized in Table S4.

**Table 2:** Nucleotide composition of the mitochondrial genome of *Liposcelis bostrychophila*.

Region	A%		T%		G%		C%		AT%		AT-skew		GC-skew	
	I^a^	II^b^	I	II	I	II	I	II	I	II	I	II	I	II
Full length	30.06	30.38	37.72	39.16	13.87	12.39	18.35	18.06	67.78	69.54	−0.113	−0.126	−0.139	−0.186
PCGs	27.42	27.37	39.55	41.68	15.23	12.77	17.80	18.18	66.97	69.05	−0.181	−0.207	−0.078	−0.175
1st codon	31.72	30.89	31.88	36.53	18.98	15.54	17.42	17.04	63.60	67.42	−0.003	−0.084	0.043	−0.046
2nd codon	18.66	21.13	48.71	46.83	14.68	13.04	17.96	19.00	67.37	67.96	−0.446	−0.378	−0.100	−0.186
3rd codon	31.88	30.09	38.07	41.69	12.04	9.72	18.01	18.60	69.95	71.79	−0.088	−0.162	−0.199	−0.311
tRNA genes	37.68	37.77	35.17	35.40	11.72	13.50	15.43	13.32	72.85	73.17	0.034	0.032	−0.137	0.007
rRNA genes	33.19	36.44	34.81	35.42	14.37	10.42	17.63	17.71	68.00	71.86	−0.024	0.014	−0.102	−0.259
Non-coding	29.88	28.43	38.20	39.50	11.90	12.58	20.02	19.49	68.09	67.93	−0.122	−0.163	−0.255	−0.215

a , Mitochondrial chromosome I;

b , Mitochondrial chromosome II; AT-skew = (A−T)/(A+T); GC-skew = (G−C)/(G+C).

### PCR verification of the multipartite mitochondrial genome of *L. bostrychophila* and quantification of the two mitochondrial chromosomes

**Figure 6:**
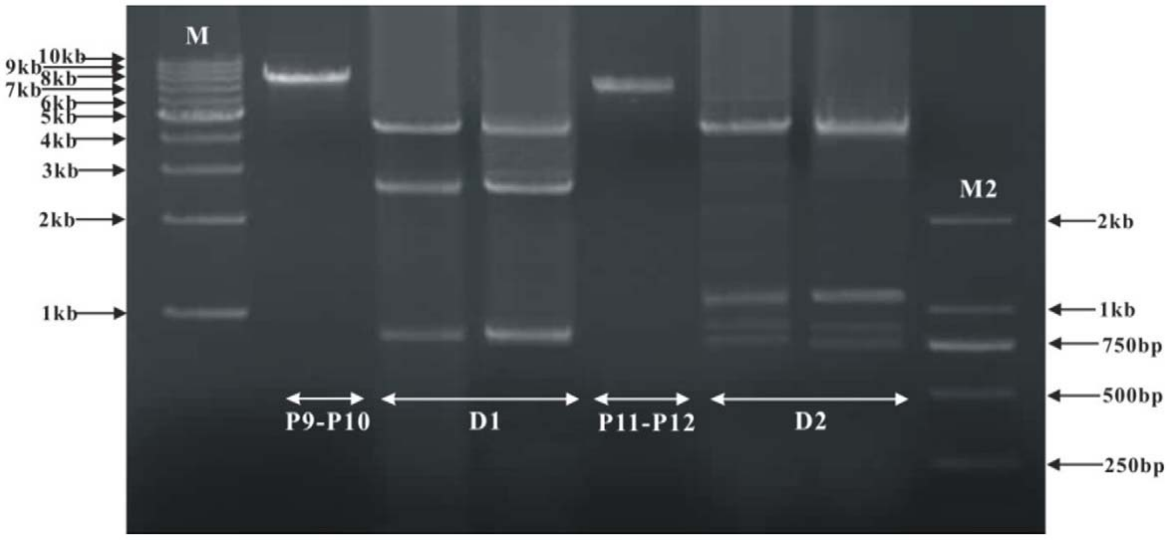
Restriction enzyme digests of long-PCR product P9–P10 and P11–P12 from Beibei strain of *Liposcelis bostrychophila*. Lane M: 1 kb marker (TaKaRa); Lane M2: DL2000 bp marker (TaKaRa). D1 represent digestion of PCR product P9–P10 which contain three fragments, 4,529 bp, 2,704 bp, and 865 bp; D2 represent digestion of PCR product P11–P12 which contain four fragments, 4,736 bp, 1,054 bp, 833 bp, and 771 bp.

**Figure 7:**
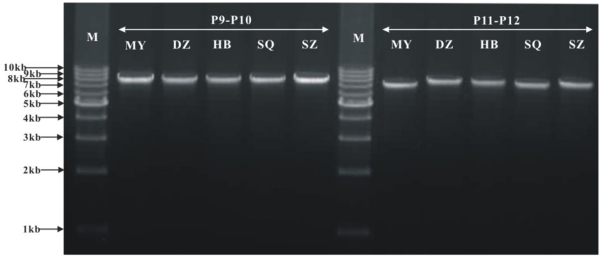
Mitochondrial genomes of other five strains of *Liposcelis bostrychophila.* See table S1 for the names of these strains.

To verify mt chromosomes I and II, we amplified the entire chromosome I with primers P9 and P10, and the entire mt chromosomes II with primers P11 and P12 (Figure 1). These PCR experiments amplified an 8.0 kb and a 7.4 kb fragment, respectively (Figure 2). We digested the 8.0 kb fragment (P9–P10) with NdeI and XbaI and obtained three DNA fragments of the sizes we expected from our sequence analyses: 865 bp, 2,704 bp, and 4,529 bp (Figure 6). We digested the 7.4 kb fragment (P11–P12) with NdeI and ApaI and obtained four DNA fragments of the sizes we expected: 771 bp, 833 bp, 1,054 bp, and 4,736 bp (Figure 6). Our PCR test and restriction enzyme digestion confirmed the size and the circular structure of mt chromosome I and mt chromosome II of *L. bostrychophila*. We also amplified these two mt chromosomes from *L. bostrychophila* collected at five other locations in China (Table S1). PCR with primer pairs P9–P10 and P11–P12 amplified fragments of the same sizes as the fragments amplified from the Beibei strain of *L. bostrychophila* (Figure 7). Our real-time PCR quantification showed unequal copy numbers between the two mt chromosomes of *L. bostrychophila*: chromosome I was nearly twice (1.869±0.077) as abundant as chromosome II (Table 3).

**Table 3:** Real-time PCR results showing the relative copy number of mt chromosome I to chromosome II.

DNA	Replicate	*Cob* (Ct)	*nad1* (Ct)	*β-Actin* (Ct)	Ratio A	Ratio B	Ratio C
SDS	1	19.08±0.014	19.95±0.057	23.38±0.050	19.698	10.778	1.828
	2	17.90±0.113	18.74±0.106	21.40±0.014	11.314	6.320	1.790
	3	18.30±0.064	19.27±0.035	22.90±0.092	24.251	12.381	1.959
Kit	1	17.76±0.085	18.65±0.156	20.87±0.028	8.634	4.659	1.853
	2	17.90±0.021	18.88±0.424	21.12±0.007	9.318	4.724	1.972
	3	17.92±0.050	18.78±0.014	21.72±0.778	13.929	7.674	1.815

Ct refers to the threshold cycle; Ratio A = 2^−[Ct*cob*-Ct*β-Actin*]^; Ratio B = 2^−[Ct*nad1*-Ct*β-Actin*]^; Ratio C = Ratio A/Ratio B = chromosome I/chromosome II; Kit: the total DNA was extracted using DNeasy Tissue kit (QIAGEN).

### Phylogeny of booklice, barklice and parasitic lice inferred from mitochondrial genome sequences

**Figure 8:**
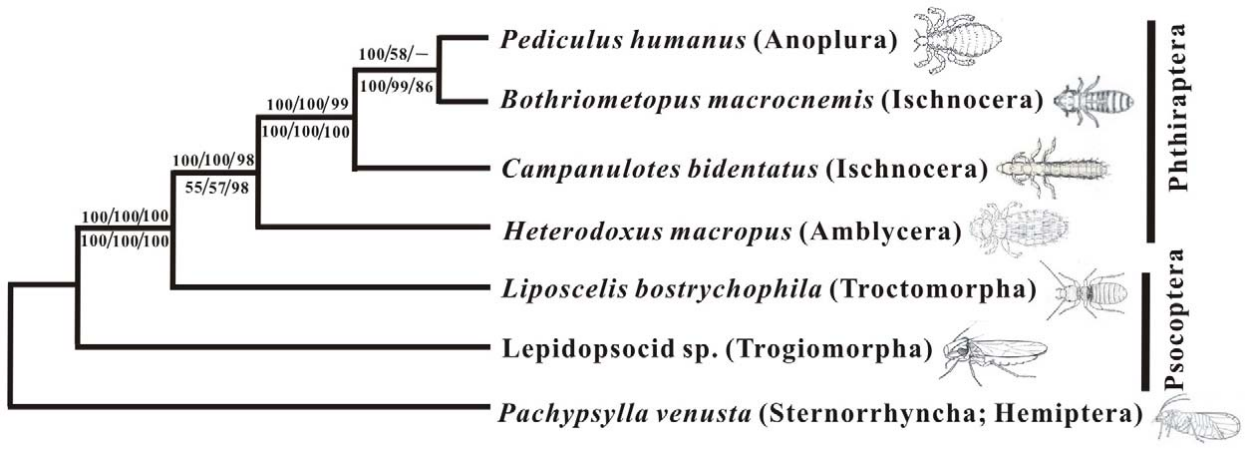
Phylogeny from mitochondrial genome sequences. Numbers above the branches show support for the phylogenies from amino acid sequences whereas numbers below the branches show support for phylogenies from nucleotide sequences: Bayesian posterior probability/ML bootstrap support values/NJ bootstrap support values. Only support above 50% is shown only.

The topologies of the phylogenetic trees inferred from the nucleotide sequences and the putative amino acid sequences were identical. In all of the phylogenetic trees constructed with different methods: 1) the parasitic lice (order Phthiraptera) were monophyletic; 2) booklice and barklice (order Psocoptera) were paraphyletic; 3) *L. bostrychophila* was the sister-group to the parasitic lice rather than the sister-group to the barklouse, Lepidopsocid sp.; and 4) one of the suborders of parasitic lice, the Ischnocera, was paraphyletic (Figure 8).

## Discussion

### Evolution of multipartite mt genomes in bilateral animals

**Figure 9:**
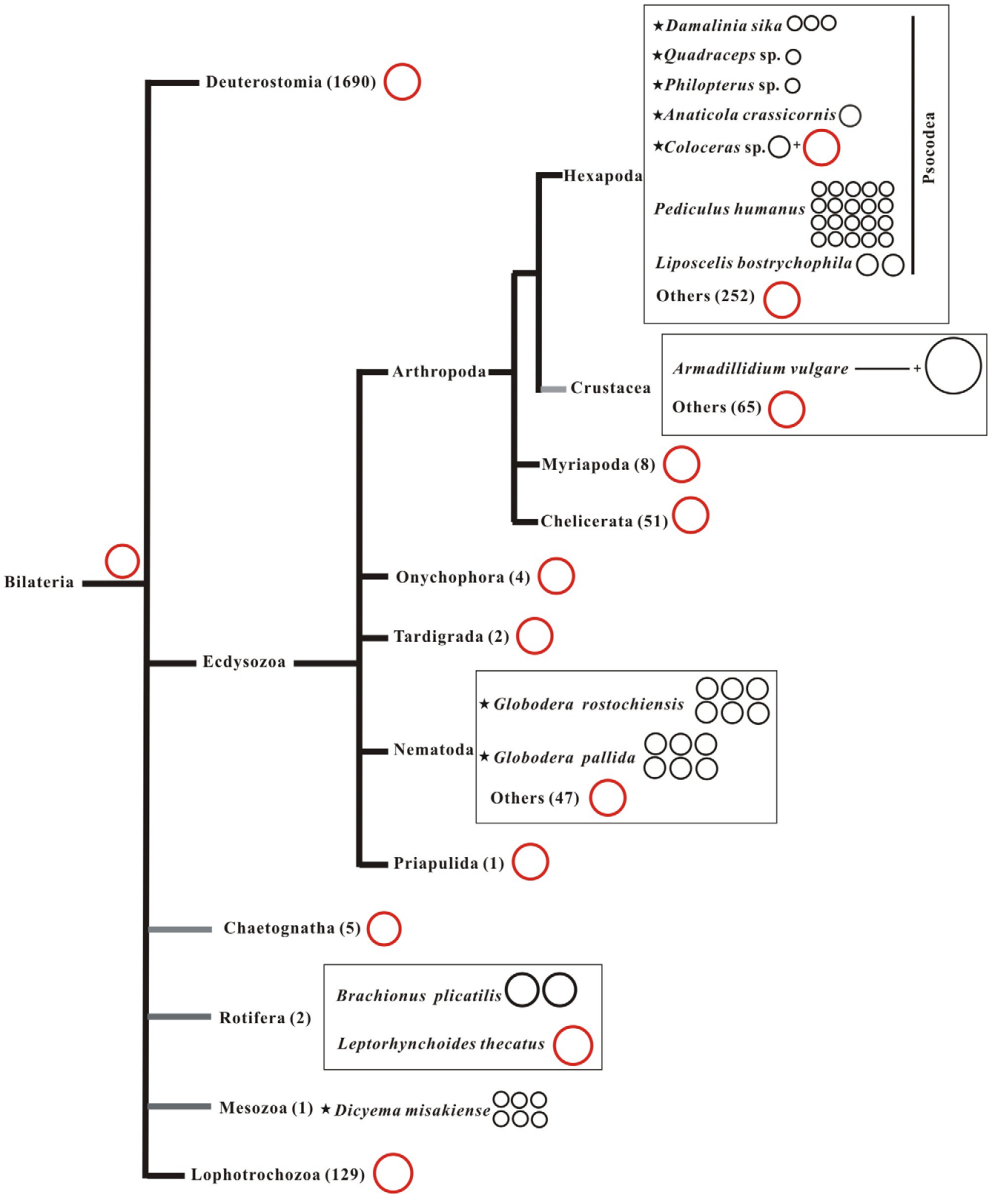
The architecture of mitochondrial chromosomes in 2,267 bilateral animals. The typical animal mitochondrial chromosome is represented as a single red color circle whereas multipartite chromosomes are represented as small circles. *Armadillidium vulgare* has a linear chromosome plus a circular chromosome. Numbers in brackets indicate the number of species for which entire genome sequences are known. Stars indicate partial mitochondrial genomes. Phylogeny from the Tree of Life Web Project (http://tolweb.org/tree/).

Multipartite mt genomes are now known from four lineages of bilateral animals: the Mesozoa, Nematode, Rotifera and Psocodea (Figure 9) [2], [4]–[7]. What drove the fragmentation of a typical mt chromosome into two or more smaller chromosomes? How could multiple small mt chromosomes replace a typical large mt chromosome? It is widely held that there has been strong selection for small and compact mt genomes in animals [54]. This selection pressure could be seen in many ways, i.e. many of the genes in the ancestral mt genome have moved to the nuclear genome or have been lost [55]; intergenic regions and introns have been lost and tRNA genes have been truncated and adjacent genes may overlap in mt chromosomes [56]–[58]. However, the typical mt chromosome of bilateral animals appears to be as compact as it can be. We propose that when mt chromosomes of different sizes co-exist, the selection pressure may favor small mt chromosomes over the typical large mt chromosome. Thus, fragmentation of mt chromosomes might be one of strategies of size-reducing for streamlining mt chromosomes and small mt chromosomes may have selective advantages over the typical large mt chromosomes. Indeed, Hayashi et al. (1991) showed that, in cultured human cells, the proportion of an 11-kb circular mtDNA increased significantly whereas the proportion of the typical 16-kb wild-type mt chromosome decreased when these two forms of mtDNA molecules co-existed for 10 weeks [59].

Mitochondrial DNA molecules smaller than the typical mt chromosomes can be generated via deletion events, as observed in nematodes [21], humans [60] and mice [61]. In plants, intramolecular homologous recombination between repeated sequences in the mt maxicircle can also generate smaller mt DNA molecules, as observed in maize [20] and cabbages [62]. In all of these cases, however, the typical mt chromosome, or the master circle, is always present. The typical mt chromosome is apparently not present in *L. bostrychophila*, the rotifer *B*. *plicatilis* [5] or the human body louse *P. humanus* [6]. We propose that the two mt chromosomes of *L. bostrychophila* were likely generated by a series of gene-deletion events; the pseudogenes in the two chromosomes were likely the remnants of such events.

### Why do the two mitochondrial chromosomes of *L. bostrychophila* have unequal copy numbers?

The copy numbers of different chromosomes of a multipartite mt genome were known previously only for the rotifer, *B. plicatilis* [5]. One of the mt chromosomes of *B. plicatilis* is four times as abundant as the other mt chromosome. Our real-time PCR showed that the two mt chromosomes of the booklouse, *L. bostrychophila*, also differ in copy number: mt chromosome I is nearly twice as abundant as mt chromosome II. If mt genes are in equal copy numbers, as in the typical mt chromosome, then we would expect that the different mt chromosomes of a multipartite mt genome be in equal copy numbers. Why are the mt chromosomes in both *L. bostrychophila* and *B. plicatilis* in unequal abundance? We speculate that different types of the chromosomes of a multipartite mt genome may be linked in some way, i.e. analogous to the network of the kinetoplast DNA [63], [64], they function and segregate as a unit, and this unit may contain different copies of each mt chromosome; or the multipartite mt chromosomes of *L. bostrychophila* may form a nucleoid structure that contains two copies of mt chromosome-I but one copy of mt chromosome-II [65]. Both of above cases will result in consistent, unequal copy numbers between different mt chromosomes.

### Phylogeny of booklice, barklice and parasitic lice

There are two hypotheses for the evolution of parasitism in the lice (super order Psocodea). First, that parasitism evolved once in the most recent common ancestor (MRCA) of the Anoplura, Rhyncophthirina, Ischnocera, and Amblycera i.e. that the order Phthiraptera is monophyletic. Second, that parasitism evolved twice: once in the MRCA of the Anoplura, Rhyncophthirina, and Ischnocera and once in the Amblycera i.e. that the order Phthiraptera is paraphyletic. Most of the molecular phylogenetic studies using sequences of gene fragments reject the first hypothesis, that parasitism evolved only once in the Psocodea, in favor of the second hypothesis, that parasitism evolved twice in these insects [22], [23], [26], [27]. Our phylogenies from mt genome sequences, however, reject the second hypothesis in favor of the first hypothesis. Our phylogenies thus indicate that parasitism evolved only once in the Psocodea and that the order Phthiraptera is monophyletic. There are also two hypotheses conceiving the Ischnocera, which is much the largest lineage of parasitic lice (over 3,120 described species on birds and mammals [66]). First, the Ischnocera is monophyletic [24] [67]. Second, the Ischnocera is paraphyletic [7]. In the present study, analyses of mt genome sequences indicate, in contrast to previous molecular and morphological studies, that the Ischnocera is paraphyletic.

## Supporting Information

Table S1 **Samples of** ***Liposcelis bostrychophila*** **used in this study.**
(DOC)

Table S2 **Mitochondrial chromosome I of** ***Liposcelis bostrychophila*** **.**
(DOC)

Table S3 **Mitochondrial chromosome II of** ***Liposcelis bostrychophila*** **.**
(DOC)

Table S4 **Codon usage for the 13 mitochondrial protein-coding genes of** ***Liposcelis bostrychophila.***
(DOC)

Figure S1 **Comparisons of Hopp/Woods hydrophilicity profiles of** ***nad4L*** **,** ***atp8*** **of** ***Liposcelis bostrychophila*** **,** ***Thrips imaginis*** **, Lepidopsocid sp.,** ***Drosophila melanogaster*** **, and** ***Homo sapiens*** **.**
(TIFF)

Figure S2 **Alignments of putative pseudogenes and putative functional genes.** Only parts of the putatively functional genes are shown.
(TIFF)
