# Supplementary material for: The Multipartite Mitochondrial Genome of Liposcelis bostrychophila: Insights into the Evolution of Mitochondrial Genomes in Bilateral Animals
Source: PLoS One. 2012 Mar 30;7(3):e33973. doi: 10.1371/journal.pone.0033973 (PMC3316519; doi:10.1371/journal.pone.0033973)
Supplement: Table S1 — Samples of Liposcelis bostrychophila used in this study. (DOC) [file pone.0033973.s001.doc]

**Table S1. Samples of *Liposcelis bostrychophila* used in this study.**

| Location | Latitude | Longitude | Collection date |
| --- | --- | --- | --- |
| Beibei, Chongqing (BB ) | 29^o^49'N | 106^o^25'E | Jun.2008 |
| Dazu, Chongqing (DZ) | 29^o^42'N | 105^o^43'E | Aug. 2008 |
| Mianyang, Sichuan (MY) | 31^o^28'N | 104^o^38'E | Aug. 2008 |
| Suizhou, Hubei (SZ) | 31^o^40'N | 113^o^21'E | Aug. 2009 |
| Huaibei, Anhui (HB) | 33^o^57'N | 116^o^48'E | Aug. 2009 |
| Shangqiu, Henan (SQ) | 34^o^25'N | 115^o^39'E | Aug. 2010 |
