# Supplementary material for: The Multipartite Mitochondrial Genome of Liposcelis bostrychophila: Insights into the Evolution of Mitochondrial Genomes in Bilateral Animals
Source: PLoS One. 2012 Mar 30;7(3):e33973. doi: 10.1371/journal.pone.0033973 (PMC3316519; doi:10.1371/journal.pone.0033973)
Supplement: Table S2 — Mitochondrial chromosome I of Liposcelis bostrychophila . (DOC) [file pone.0033973.s002.doc]

**Table S2. Mitochondrial chromosome I of *Liposcelis bostrychophila*.**

| Gene^a^ | Region | Size | INC^b^ | AT% | AT-skew^c^ | GC-skew^c^ | | Start codon | Stop codon |
| --- | --- | --- | --- | --- | --- | --- | --- | --- | --- |
| ***NCRI*** | 1-6 | 6 | 0 | 100 | 0 |  |  | |  |
| ***cox1*** | 7-1548 | 1542 | 6 | 62.84 | -0.187 | -0.089 | ATC | | TAA |
| ***trnD*** | 1544-1602 | 59 | -5 | 76.27 | -0.111 | 0.286 |  | |  |
| ***nad4L*** | 1604-1849 | 246 | 1 | 74.39 | -0.104 | 0.111 | ATT | | TAA |
| ***trnS1*** | 1871-1927 | 57 | 21 | 59.65 | 0.059 | -0.304 |  | |  |
| ***nad2*** | 1936-2787 | 852 | 8 | 72.65 | -0.212 | -0.167 | ATT | | TAA |
| ***NCRI-1*** | 2788-2843 | 56 | 0 | 75.00 | 0.048 | 0 |  | |  |
| ***trnT*** | 2844-2903 | 60 | 0 | 80.00 | -0.083 | 0.333 |  | |  |
| ***trnR*** | 2922-2972 | 51 | 18 | 64.71 | -0.152 | 0.222 |  | |  |
| ***NCRI-2*** | 2973-3024 | 52 | 0 | 63.46 | 0.212 | -0.368 |  | |  |
| ***P-nad4*** | 3025-3240 | 216 | 0 | 74.07 | -0.163 | -0.321 |  | |  |
| ***trnW*** | 3275-3336 | 62 | 34 | 82.26 | -0.059 | 0.091 |  | |  |
| ***cob*** | 3351-4403 | 1053 | 14 | 65.72 | -0.173 | -0.125 | ATT | | TAA |
| ***nad6*** | 4400-4846 | 447 | -4 | 72.26 | -0.238 | 0.258 | ATT | | TAA |
| ***P-nad5*** | 4861-5067 | 207 | 14 | 61.35 | -0.228 | -0.300 |  | |  |
| ***trnL1*** | 5087-5147 | 61 | 19 | 77.05 | 0.149 | 0.286 |  | |  |
| ***trnI*** | 5144-5207 | 64 | -4 | 71.88 | 0 | 0.333 |  | |  |
| ***trnC*** | 5219-5275 | 54 | 11 | 62.96 | -0.059 | 0.100 |  | |  |
| ***IR*** | 5280-6224 | 945 | 7 | 69.31 | -0.108 | -0.166 |  | |  |
| ***NCRI-3*** | 5280-5406 | 127 | 0 | 66.14 | -0.095 | -0.395 |  | |  |
| ***trnA*** | 5407-5472 | 66 | 0 | 77.27 | -0.059 | 0.067 |  | |  |
| ***NCRI-4*** | 5473-5957 | 485 | 0 | 66.39 | -0.242 | -0.264 |  | |  |
| ***trnE*** | 5958-6011 | 54 | 0 | 81.48 | 0.273 | 0 |  | |  |
| ***trnM*** | 6009-6068 | 60 | -3 | 71.67 | 0.163 | 0.176 |  | |  |
| ***NCRI-5*** | 6069-6224 | 156 | 0 | 73.08 | -0.018 | 0.190 |  | |  |
| ***rrnS*** | 6225-6899 | 675 | 0 | 68.00 | -0.024 | -0.102 |  | |  |
| ***cox2*** | 6900-7556 | 657 | 0 | 65.60 | -0.114 | -0.062 | ATA | | TAA |
| ***trnS2*** | 7561-7623 | 63 | 4 | 65.08 | -0.073 | -0.182 |  | |  |
| ***trnV*** | 7622-7685 | 64 | -2 | 67.19 | -0.023 | 0.048 |  | |  |
| ***trnG*** | 7687-7747 | 61 | 1 | 80.33 | 0.020 | -0.167 |  | |  |
| ***cox3*** | 7748-8530 | 783 | 0 | 66.41 | -0.192 | -0.125 | ATA | | TAA |

^a^Genes underlined are on the majority strand; genes not underlined are on the minority strand. ^b^INC: intergenic nucleotides; positive values indicate gaps and negative values indicate overlapped nucleotides between adjacent genes. ^c^AT-skew = (A-T)/(A+T), GC-skew = (G-C)/(G+C).
