# Supplementary material for: The Multipartite Mitochondrial Genome of Liposcelis bostrychophila: Insights into the Evolution of Mitochondrial Genomes in Bilateral Animals
Source: PLoS One. 2012 Mar 30;7(3):e33973. doi: 10.1371/journal.pone.0033973 (PMC3316519; doi:10.1371/journal.pone.0033973)
Supplement: Table S3 — Mitochondrial chromosome II of Liposcelis bostrychophila . (DOC) [file pone.0033973.s003.doc]

**Table S3. Mitochondrial chromosome II of *Liposcelis bostrychophila*.**

| Gene^a^ | Region | Size | INC^b^ | AT% | AT-skew^c^ | GC-skew^c^ | Start codon | Stop codon |
| --- | --- | --- | --- | --- | --- | --- | --- | --- |
| ***NCRII-1*** | 1-77 | 77 | 0 | 70.13 | -0.037 | -0.130 |  |  |
| ***IR*** | 78-1022 | 945 | 0 | 70.31 | -0.022 | -0.053 |  |  |
| ***NCRII-2*** | 78-204 | 127 | 0 | 66.14 | -0.095 | -0.395 |  |  |
| ***trnA*** | 205-270 | 66 | 204 | 81.90 | 0.000 | -0.018 |  |  |
| ***NCRII-3*** | 271-755 | 485 | 0 | 66.39 | -0.242 | -0.264 |  |  |
| ***trnE*** | 756-809 | 54 | 0 | 89.72 | -0.243 | 0.030 |  |  |
| ***trnM*** | 807-866 | 60 | -3 | 87.54 | 0.104 | 0.160 |  |  |
| ***NCRII-4*** | 867-1022 | 108 | 0 | 72.22 | -0.103 | 0.400 |  |  |
| ***trnK*** | 975-1035 | 61 | 0 | 68.85 | 0.314 | -0.263 |  |  |
| ***NCRII-5*** | 1036-1232 | 197 | 0 | 62.94 | -0.065 | -0.205 |  |  |
| ***trnP*** | 1233-1294 | 62 | 0 | 77.42 | 0.677 | 0.286 |  |  |
| ***trnQ*** | 1289-1351 | 63 | -6 | 73.02 | 0.533 | 0.176 |  |  |
| ***nad3*** | 1352-1693 | 342 | 0 | 71.35 | 0.286 | 0.082 | ATG | TAG |
| ***trnL2*** | 1694-1754 | 61 | 0 | 73.77 | 0.548 | 0.125 |  |  |
| ***P-nad5*** | 1795-1902 | 108 | 40 | 74.07 | 0.286 | -0.429 |  |  |
| ***NCRII-6*** | 1903-1975 | 73 | 0 | 82.19 | -0.233 | -0.846 |  |  |
| ***P-cox3*** | 1976-2297 | 322 | 0 | 67.39 | 0.108 | -0.105 |  |  |
| ***rrnL*** | 2298-3381 | 1084 | 0 | 71.86 | 0.014 | -0.259 |  |  |
| ***trnY*** | 3382-3441 | 60 | 0 | 61.67 | 0.188 | -0.130 |  |  |
| ***trnF*** | 3451-3511 | 61 | 9 | 73.77 | 0.692 | 0.500 |  |  |
| ***nad5*** | 3526-5094 | 1569 | 14 | 69.28 | 0.227 | -0.178 | ATT | TAG |
| ***nad4*** | 5095-6303 | 1209 | 0 | 70.31 | 0.213 | -0.203 | ATC | TAA |
| ***nad1*** | 6303-7176 | 874 | -1 | 67.51 | 0.127 | -0.232 | ATC | T |
| ***atp8*** | 7177-7332 | 156 | 0 | 67.95 | 0.215 | -0.240 | GTG | TAG |
| ***atp6*** | 7298-7933 | 636 | -35 | 67.30 | 0.179 | -0.144 | ATA | TAA |

^a^Genes underlined are on the majority strand; genes not underlined are on the minority strand. ^b^INC: intergenic nucleotides; positive values indicate gaps and negative values indicate overlapped nucleotides between adjacent genes. ^c^AT-skew = (A-T)/(A+T), GC-skew = (G-C)/(G+C).
