# Supplementary material for: The Multipartite Mitochondrial Genome of Liposcelis bostrychophila: Insights into the Evolution of Mitochondrial Genomes in Bilateral Animals
Source: PLoS One. 2012 Mar 30;7(3):e33973. doi: 10.1371/journal.pone.0033973 (PMC3316519; doi:10.1371/journal.pone.0033973)
Supplement: Table S4 — Codon usage for the 13 mitochondrial protein-coding genes of Liposcelis bostrychophila. (DOC) [file pone.0033973.s004.doc]

**Table S4. Codon usage for the 13 mitochondrial protein-coding genes of *Liposcelis bosrychophila*.**

| Codon  (amino acid) | N | % | RSCU | Codon | N | % | RSCU | Codon | N | % | RSCU | Codon | N | % | RSCU |
| --- | --- | --- | --- | --- | --- | --- | --- | --- | --- | --- | --- | --- | --- | --- | --- |
| UUU(F) | 297 | 8.6 | 1.55 | UCU(S) | 109 | 3.15 | 2.1 | UAU(Y) | 93 | 2.69 | 1.35 | UGU(C) | 33 | 0.96 | 1.61 |
| UUC(F) | 86 | 2.49 | 0.45 | UCC(S) | 41 | 1.17 | 0.79 | UAC(Y) | 45 | 1.3 | 0.65 | UGC(C) | 8 | 0.23 | 0.39 |
| UUA(L) | 195 | 5.64 | 2.13 | UCA(S) | 98 | 2.84 | 1.89 | UAA(*) | 15 | 0.43 | 1.36 | UGA(W) | 65 | 1.89 | 1.49 |
| UUG(L) | 43 | 1.24 | 0.47 | UCG(S) | 19 | 0.55 | 0.37 | UAG(*) | 7 | 0.2 | 0.64 | UGG(W) | 22 | 0.64 | 0.51 |
| CUU(L) | 131 | 3.79 | 1.43 | CCU(P) | 53 | 1.53 | 1.8 | CAU(H) | 40 | 1.16 | 1.16 | CGU(R) | 12 | 0.35 | 1.14 |
| CUC(L) | 54 | 1.56 | 0.59 | CCC(P) | 32 | 0.93 | 1.08 | CAC(H) | 29 | 0.84 | 0.84 | CGC(R) | 9 | 0.26 | 0.86 |
| CUA(L) | 92 | 2.66 | 1 | CCA(P) | 31 | 0.9 | 1.05 | CAA(Q) | 37 | 1.07 | 1.35 | CGA(R) | 17 | 0.49 | 1.62 |
| CUG(L) | 35 | 1.01 | 0.38 | CCG(P) | 2 | 0.06 | 0.07 | CAG(Q) | 18 | 0.52 | 0.65 | CGG(R) | 4 | 0.12 | 0.38 |
| AUU(I) | 221 | 6.4 | 1.34 | ACU(T) | 57 | 1.65 | 1.54 | AAU(N) | 95 | 2.75 | 1.31 | AGU(S) | 19 | 0.55 | 0.37 |
| AUC(I) | 110 | 3.18 | 0.66 | ACC(T) | 38 | 1.1 | 1.03 | AAC(N) | 50 | 1.45 | 0.69 | AGC(S) | 23 | 0.67 | 0.44 |
| AUA(M) | 137 | 3.97 | 1.43 | ACA(T) | 44 | 1.27 | 1.19 | AAA(K) | 91 | 2.63 | 1.53 | AGA(S) | 72 | 2.08 | 1.39 |
| AUG(M) | 54 | 1.56 | 0.57 | ACG(T) | 9 | 0.26 | 0.24 | AAG(K) | 28 | 0.81 | 0.47 | AGG(S) | 34 | 0.98 | 0.66 |
| GUU(V) | 94 | 2.72 | 1.9 | GCU(A) | 43 | 1.24 | 1.65 | GAU(D) | 46 | 1.33 | 1.46 | GGU(G) | 30 | 0.87 | 0.74 |
| GUC(V) | 33 | 0.96 | 0.67 | GCC(A) | 30 | 0.87 | 1.15 | GAC(D) | 17 | 0.49 | 0.54 | GGC(G) | 25 | 0.72 | 0.61 |
| GUA(V) | 49 | 1.42 | 0.99 | GCA(A) | 28 | 0.81 | 1.08 | GAA(E) | 49 | 1.42 | 1.34 | GGA(G) | 53 | 1.53 | 1.3 |
| GUG(V) | 22 | 0.64 | 0.44 | GCG(A) | 3 | 0.08 | 0.12 | GAG(E) | 24 | 0.69 | 0.66 | GGG(G) | 55 | 1.59 | 1.35 |

Note: The 13 mitochondrial protein-coding genes of *L. bostrychophila* have 3,455 codons in total. N = frequency of each codon. % = N/3455. RSCU, Relative synonymous codon usage (RSCUi = *X*i / ∑∑ *X*I /n, *X*i is the number of times the ith codon has been used for a given amino acid, and n is the number of synonymous codons for that amino acid.)
