# Supplementary figures and images for: The Multipartite Mitochondrial Genome of Liposcelis bostrychophila: Insights into the Evolution of Mitochondrial Genomes in Bilateral Animals
Source: PLoS One. 2012 Mar 30;7(3):e33973. doi: 10.1371/journal.pone.0033973 (PMC3316519; doi:10.1371/journal.pone.0033973)

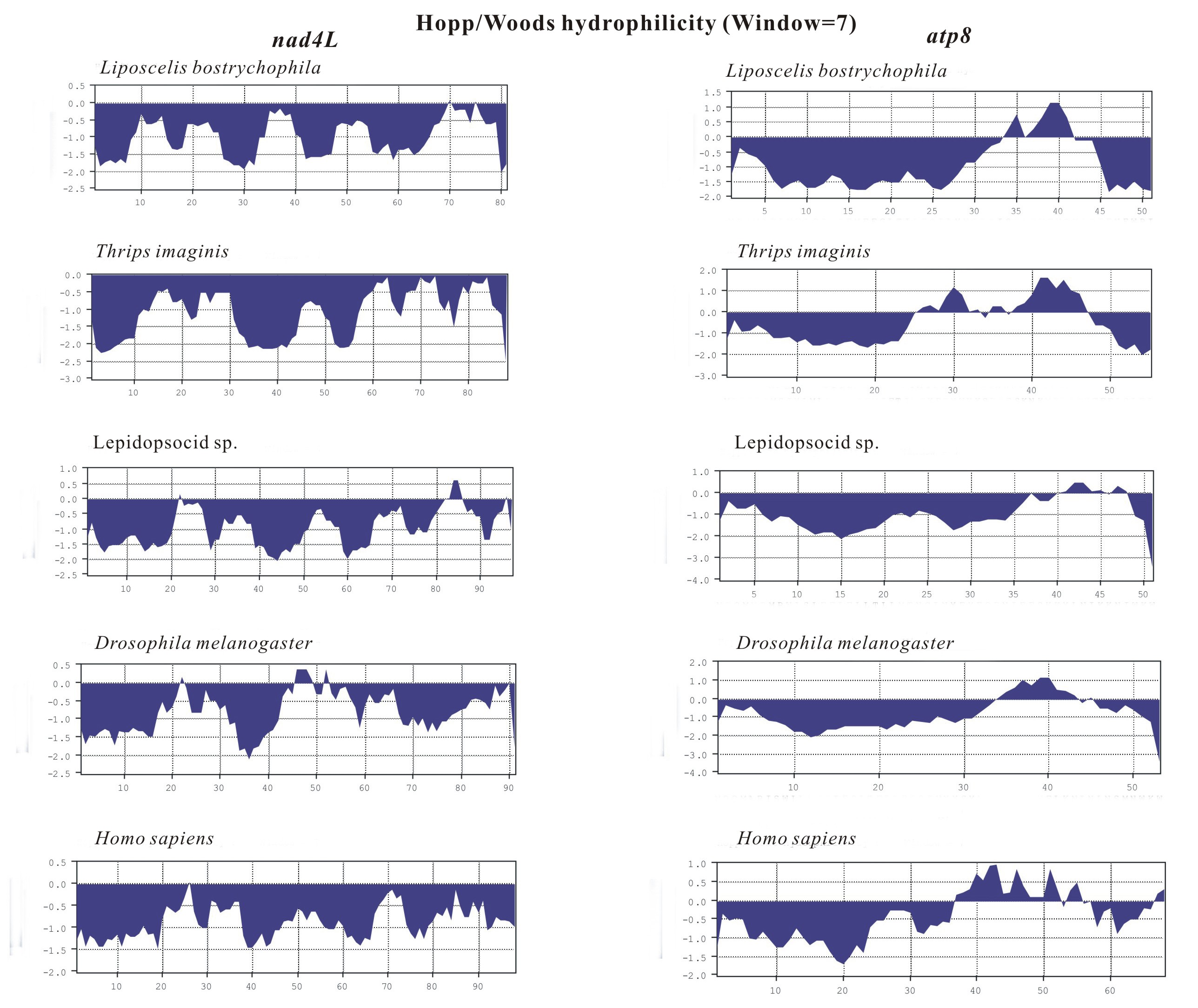

Supplement: Figure S1 — Comparisons of Hopp/Woods hydrophilicity profiles of nad4L , atp8 of Liposcelis bostrychophila , Thrips imaginis , Lepidopsocid sp., Drosophila melanogaster , and Homo sapiens . (TIFF) [file pone.0033973.s005.tiff]

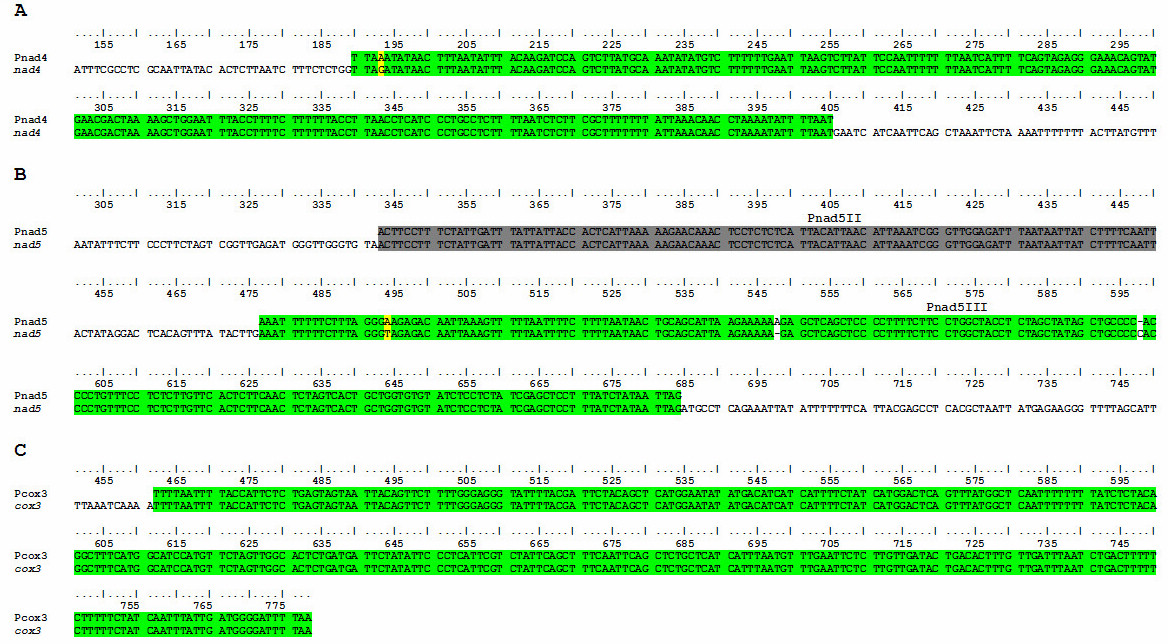

Supplement: Figure S2 — Alignments of putative pseudogenes and putative functional genes. Only parts of the putatively functional genes are shown. (TIFF) [file pone.0033973.s006.tiff]
